# Supervision and MFT burnout: overcoming the challenges therapists face in the workplace

**DOI:** 10.3389/fpsyg.2015.01644

**Published:** 2015-10-27

**Authors:** Gilbert E. Franco

**Affiliations:** Graduate Behavioral Sciences, Southern California SeminaryEl Cajon, CA, USA

**Keywords:** clinical supervision, work environment, therapist, marriage and family therapists, licensed professional clinical counselors, psychologist, licensed clinical social workers, burnout

Most of us know that feeling. As we lay in our beds we close our eyes, but no matter how hard we try, we can't stop thinking about that one client. We ask ourselves whether we could have prevented them from going into crisis and threatening to commit suicide. We wonder whether having them hospitalized was the right choice.

How about our assessment that morning? That poor woman just found out that her 15 year old grandson was using drugs since he was 10. Or maybe they are thoughts of that couple we saw yesterday. They are triangulating their 5 year old child into their relationship, but have difficulty acknowledging it no matter how many times we point it out.

These images flood our mind until 1 day, we just stop caring. Our minds go numb. We are human, after all, and can only take so much. We burn out.

Burnout is may arise as a result of prolonged time of stress that is a result of someone not achieving their goals (van Dam et al., 2011). For example, therapists working at community mental health agencies may experience burnout as a result of not being able to meet monthly productivity standards. After months of not meeting their productivity, the therapist may simply just give up. This can be a problem because it can be difficult to recover from burnout.

Recovery from burnout can be difficult. In a study conducted on individuals with burnout, van Dam et al. (2011) found that even when motivational interventions have been implemented, individuals with burnout were averse to expending more effort and did not improve their performance. van Dam et al.'s findings suggest that the prevention of burnout may be an effective course of action to prevent the challenges associated with treating burnout after it has occurred. In order to prevent burnout, though, one must know what burnout is.

Studies have discussed the concept of burnout as a process that involves failure, wearing out, or becoming exhausted due to excessive demands on a person's resources, energy, and strength (Freudenberger, 1974; Bianchi et al., 2013; Cieslak et al., 2014; Shin et al., 2014). Freudenberger asserts that there are both behavioral and physical symptoms of burnout (Freudenberger, 1974). Symptoms such as exhaustion and sleepiness comprise of physical symptoms of burnout (Freudenberger, 1974; Cieslak et al., 2014; Shin et al., 2014). Behavioral symptoms of burnout can include difficulty to holding in feelings and verbalizing negative attitudes (Freudenberger, 1974; Shin et al., 2014). Freudenberger further asserts that people working long hours in jobs with little compensation are prone to burnout and points out therapeutic communities, free clinics, and crisis intervention centers as examples of settings containing jobs with high burnout (Freudenberger, 1974).

More recent studies argue that professionals working in mental health are at a high risk of developing burnout (Finnøy, 2000; Jenaro et al., 2007; Valenti et al., 2014). Green et al. (2014) assert that burnout adversely impacts client quality of care. Thomas et al. (2014) conducted a study on job burnout by administering a 13 item burnout scale to a convenience sample of 288 human service workers. They found that caseload size was the most significant predictor of human service worker burnout (Thomas et al., 2014). Marriage and family therapists (MFTs) are also at risk.

Rosenberg and Pace (2006) conducted a study on MFTs and burnout. Rosenberg and Pace found that MFTs whom worked in community mental agencies had higher burnout rates than MFTs working in private practice (Rosenberg and Pace, 2006). Program managers and clinical supervisors of MFTs working in community mental health agencies may have to pay special attention to their supervisee's status at work and provide the resources necessary to enable their supervisees to address or prevent symptoms of burnout.

A key to preventing burnout in MFTs is their work environment. MFTs working as clinicians in community agencies have to contend with expectations, rules, policies, large caseloads, administrative responsibilities, and limited salaries (Rosenberg and Pace, 2006). MFTs may be caught in a struggle between fulfilling their responsibilities to their clients and their responsibilities with the agency (Rosenberg and Pace, 2006).

To illustrate this, let us imagine a situation where you are a community mental health clinician. It is the end of the month and you are 5 h short of your productivity standard for the month. You did not make your productivity standard for the last 2 months and your program manager stated that you must meet productivity standards this month. You had six scheduled appointments that day, but two did not show and now you are still behind for the month. What do you do? Do you frantically call clients in order to make up for your lost time? Is it ethical to do so?

As MFT supervisors, it is important to remember our skills in empathy that we learned as clinicians and ask ourselves, are we creating work environments that promote burnout in our therapists? Rosenberg and Pace assert that, through education, supervision, and self-awareness, there should be increased awareness in the signs and symptoms of burnout through education (Rosenberg and Pace, 2006). Additionally, they assert that self-care measures and collegial supports are important for preventing the negative effects of burnout (Rosenberg and Pace, 2006).

Supervisors can educate themselves and their clinicians by further researching burnout and its detrimental effects to their staff. Supervisors can discuss and promote therapist self-awareness and self-care during supervision. Supervisors can also promote collegial supports during treatment team meetings and by having team-building exercises. Instead of increasing stress and chances of burnout by writing-up low performing therapists, supervisors can work together with therapists and provide support in helping them achieve their productivity goals. By addressing and preventing therapist burnout, supervisors can help create a positive work environment that will ultimately, benefit the clients that the agency serves.

## Conflict of interest statement

The author declares that the research was conducted in the absence of any commercial or financial relationships that could be construed as a potential conflict of interest.
